# The magnitude of correlation between deadlift 1RM and jumping performance is sports dependent

**DOI:** 10.3389/fspor.2024.1345213

**Published:** 2024-01-15

**Authors:** Stephan Schiemann, Michael Keiner, Klaus Wirth, Lars H. Lohmann, Carl-Maximilian Wagner, David G. Behm, Konstantin Warneke

**Affiliations:** ^1^ Insitute of Exercise, Sport and Health, Leuphana University Lüneburg, Lüneburg, Germany; ^2^ Department for Sport Science, German University of Health and Sport, Ismaning, Germany; ^3^ Department for Sport Science, University of Applied Sciences Wiener Neustadt, Wiener Neustadt, Austria; ^4^ Institute of Movement Science and Exercise Physiology, Friedrich Schiller University Jena, Jena, Germany; ^5^ School of Human Kinetics and Recreation, Memorial University of Newfoundland, St. John's, NL, Canada; ^6^ Institute of Sport Science, Alpen-Adria University Klagenfurt, Klagenfurt am Wörthersee, Austria

**Keywords:** maximum strength, deadlift, vertical jump, athletes, basketball, soccer, powerlifting

## Abstract

**Introduction:**

Based on the assumption of maximal strength as a basic ability, several studies show a high influence of maximum strength on jumping performance in several sport athletes. However, there is a wide range of correlations from *r* = 0.17–0.9 between squat 1RM and jumping performance in different sports. Additionally, there are only a few studies investigating the influence of deadlift one repetition maximum (1RM) on jumping performance. Thus, this study aimed to investigate the correlations between 1RM in the deadlift on jumping performance using the countermovement jump height (CMJ) and squat jump height (SJ) considering different sports.

**Methods:**

103 athletes with experience in the deadlift from soccer, basketball, American football, powerlifting as well as participants from different sports without any deadlift experience (control group) were included to this study.

**Results:**

Overall statistics showed a significant moderate influence of deadlift 1RM (*r* = 0.301–0.472) on jumping performance. However, subgroup analysis showed no significant correlation between deadlift 1RM and jumping performance in control participants, while moderate correlations could be detected in powerlifters (*r* = 0.34–0.39), soccer players (*r* = 0.437–0.46), American football players (0.584–0.62) and high correlations in basketball players (*r* = 0.809–0.848) showing significant influence of type of sport on correlations between deadlift maximum strength and jumping performance.

**Discussion:**

Presented results underline movement velocity- and task specificity of strength training routines which is discussed in the light of the respective sports.

## Introduction

Maximal strength can be assumed to be a basic component influencing human movements with an undisputable influence on speed strength performance (1). Accordingly, a vast amount of studies point out the benefits of maximum strength in the lower extremity on jumping and sprinting performance in team sports (2, 3) such as soccer (4–6), handball (7), volleyball (8) or basketball (9–11). Since vertical jump performance is paramount for on-field performance in many of these team sports, most performance test batteries measure the vertical jump ability quantified as the jumping height usually via the countermovement jump (CMJ) or squat jump (SJ) (4, 11–13). Furthermore, SJ and CMJ are frequently used as predictors for speed strength performance and more specifically for rate of force development (1, 14).

Numerous studies reported the influence of high levels of maximal strength performance mostly measured via the one repetition maximum (1RM) or maximal isometric strength. Wisloff et al. (15) determined correlations between one repetition maximum (1RM) squat and vertical jump performance of *r* = 0.71 in 17 male elite soccer players, which was confirmed by Möck and colleagues (16) and Comfort et al. (17) who found similar significant correlations between squat strength and jumping performance of *r* = 0.79 in soccer players. While Requena et al. (18) showed the half squat to be moderately related to the CMJ and SJ performance of *r* = 0.5 in 21 male soccer players Boraczynski et al. (19) found small, but significant correlations of only *r* = 0.39 between 1RM half squat and CMJ jumping height in 25 soccer players. While the lowest relationship between maximum strength and jumping performance was determined by Requena et al. (20) with a trivial correlation between 1RM squat and CMJ performance (*r* = 0.17; *p* > 0.05) in 21 semi-professional sprinters. Warneke et al. (11) achieved the highest correlations with *r* = 0.89–0.91, using the deep front squat (11) in 42 elite basketball players (*r* = 0.89–0.91).

Apart from the squat, current literature suggests more exercises leading to similar activation of the lower extremity muscles such as step-ups, lunges and deadlifts (21, 22). Both, the squat and the deadlift permit the use of high loads (23–25) with a high relationship between those two exercises (11). Accordingly, comparable to the squat, evidence underlines the potential of using the deadlift to improve jumping performance (11, 26), however, while direct evidence calculating correlations is scarce.

Furthermore, all listed studies were performed with athletes from different sports backgrounds, thus making it difficult to isolate the influence of maximum strength on jumping performance, as learning effects (27). As movement velocity specificity (28) suggests that the training and task activity should closely match, it could be speculated that athletes who are accustomed to jumping may show higher transferability of maximum strength to jumping performance. However, in many periodized training programs, exercises such as deadlifts and squats are performed during the maximum strength and hypertrophy phases, which is then followed by power mesocycles with exercises such as plyometrics. The extent of transferability from deadlifts to jumping and sprinting performance would be important for designing effective training programs (29, 30). Even if there is a sports dependency between maximum strength in the lower extremity and jumping performance, no studies could be found comparing the impact of 1RM performance on vertical jumping height, considering the sport specific conditional profile.

Accordingly, it is hypothesized that the influence of maximum strength in the deadlift on jumping performance depends on type of training of athletes. Thus, among other factors such as training level of the participants, the daily training routine (i.e., inclusion of sprints, jumps, explosive movements in their sport or training) might also explain the range of correlation coefficients ranging from 0.17 (20) to *r* = 0.89 (11). Consequently, the aim of this study was to investigate the influence of 1RM in the deadlift on jumping performance in consideration of different sport backgrounds.

## Methods

### Experimental approach to the Problem

To answer the research question 103 participants from different sports were included to the study. To investigate the sport specific correlation between maximum strength and jumping performance, athletes from different sports with different conditional requests were tested in the CMJ, SJ and 1RM deadlift. To investigate the role of regular jump training, a group of elite powerlifters was included to the study, to provide high maximum strength values but without including jumping exercises to their common training routine. All participants were familiar with the 1RM deadlift testing, except of the control group consisting of participants from different sports without any experience in deadlift.

### Subjects

The sample size was calculated using G-Power 3.1. A total sample size of at least 42 participants was determined, assuming an effect size of *r* = 0.5. 103 male participants (age: 23.4 ± 3.6, height: 182.5 ± 8.4 cm, weight: 87.5 ± 7.4 kg) from basketball (*n* = 24, age: 22.5 ± 1.3 years, height: 1.93 ± 0.11 m, weight: 92.5 ± 4.1 kg), soccer (*n* = 23, age: 20.3 ± 3.3 years, height: 1.83 ± 2.2 m, weight: 83.0 ± 3.1 kg), powerlifting (*n* = 29, age: 25.3 ± 4.7 years, height: 1.81 ± 3.4 m, weight: 90.3 ± 6.4 kg), American football (*n* = 11, age:23.4 ± 3.5, height: 182.3 ± 2.4 cm, weight: 86.3 ± 3.2) and participants from different sports without prior experience in strength training in addition to their sport specific training routine (*n* = 21, age: 21.2 ± 2.3 years, height: 179.5 ± 5.4 cm, weight: 82.4 ± 4.9 kg) were recruited. All athletes competed in high level German leagues in basketball, soccer and American football. The powerlifters were recruited from a national deadlift contest. Participants from different sports without any previous experience in deadlift training were classified as controlled condition for this study. Study design was approved by the institutional review board (Carl von Ossietzky University Oldenburg, No. Drs.EK/2022/027-01). The study was performed with human participants in accordance with the Helsinki Declaration.

### Testing of jumping performance

Before testing, a warm-up was performed using 3–5 min of self-determined low intensity jogging and 3 sets with 10 repetitions of full range of motion bodyweight squats. To evaluate vertical jump performance a force plate (BP600900-4000, AMTI, Watertown, MA) with a surface area of 50 × 60 cm and a measurement range of ± 10,000N with a sampling rate of 1,000 Hz was used. Jumping height was determined using the flight time in the CMJ and SJ. Two highly experienced investigators who were familiar with jumping diagnostics focused on the movement execution (e.g., no bending of the knees during the landing phase). Participants were instructed to keep their hands on the hips (akimbo). To consider the jumping test for further processing within the study, both investigators had to confirm the correct movement execution independently from each other to confirm the described requirements. For the SJ, participants had to hold the starting position using an 80–100° starting knee angle for at least one second, while for the CMJ, the participants were instructed to use a self-selected countermovement depth to reach maximal jumping height. Movement execution was further checked using the time-force curve. All participants performed three attempts with one-minute rest in-between. Jumping tests can be stated as highly reliable with an inter-day reliability of ICC = 0.91–0.93 and high intra-day reliability of ICC = 0.97–0.99 (11, 27). The highest reached value was used for further calculation.

### Testing of the one repetition maximum in the deadlift

**Figure 1:**
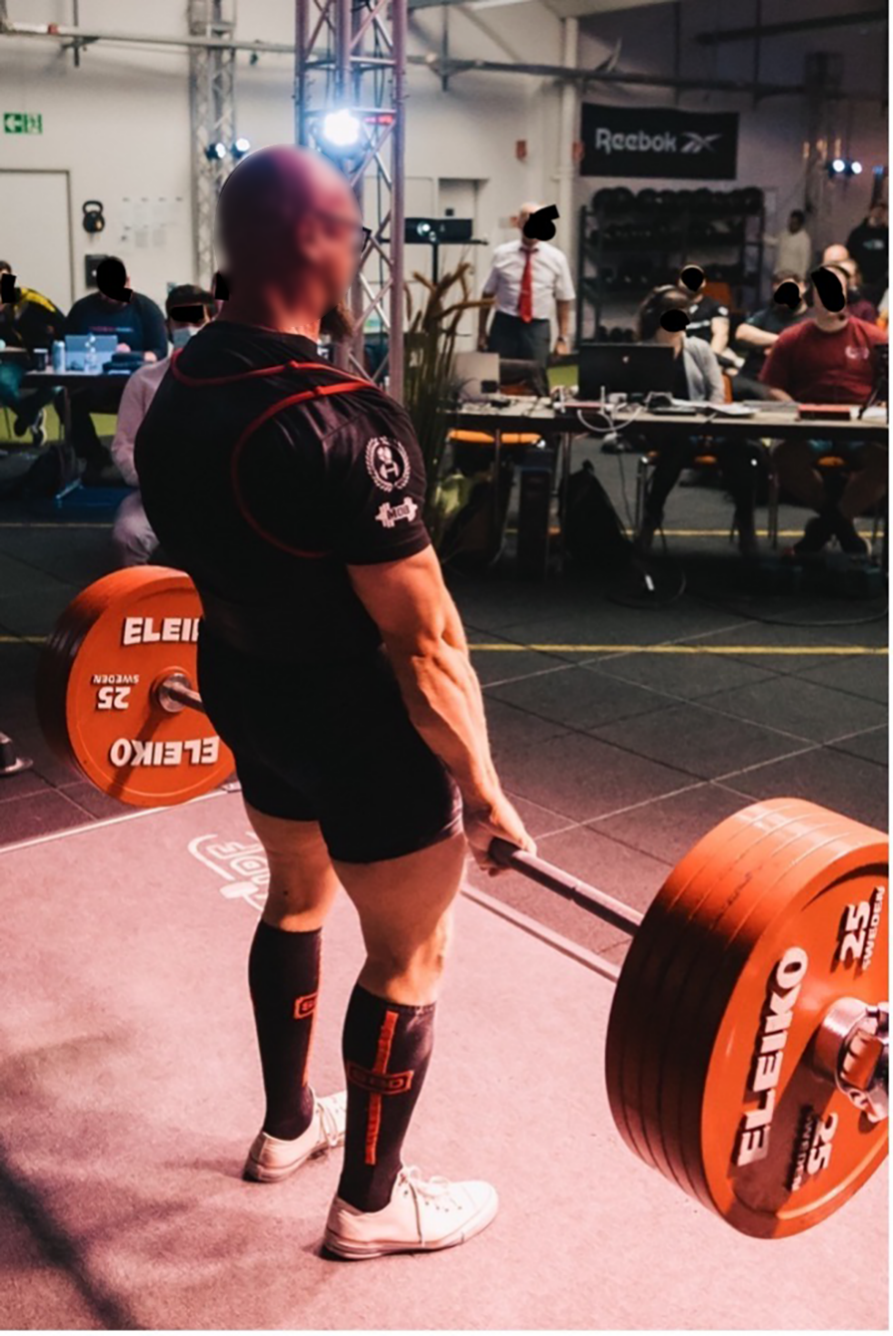
Professional powerlifter performing a 275 kg lift with a bodyweight of <80 kg.

The 1RM in the deadlift was determined performing a conventional deadlift with the favored stance and grip width, using calibrated weights from official powerlifting competitions. Participants were instructed to perform a specific and individual warm-up as mostly used in training routines if the deadlift is usually integrated in the training program. Participants of the untrained group were instructed by the investigator starting with technique check with the unloaded bar. If possible, the participant was instructed to add weight to the bar, which was dependent on the movement execution of the participant. All participants were familiar from regular strength and conditioning training with the conventional deadlift. The deadlift in all groups was rated by two independent raters and was only determined valid if a full hip and knee extension was reached at the end of the movement. An independent, experienced jury evaluated the 1RM trials of the elite powerlifters during a competition (see Figure 1). Furthermore, measurement ended after failing the same load twice or upon rounding in the lower back.

### Statistical analysis

Data were analyzed using SPSS 28.0 (IBM, Ehningen, Germany). The significance level for all statistical tests was set at *p* < 0.05. The descriptive statistics for all measurements are presented as the mean ± standard (M ± SD) deviation. Intra-session reliability for the jump performance was done for the best and the second-best trial using intraclass correlation coefficient (ICC) with 95% confidence interval (95% CI) and the coefficient of variability (31). The Kolmogorov-Smirnov Test was used to evaluate normal distribution of data. Correlations for overall statistics and subgroup analyses were determined using bivariate Pearson correlation coefficients (r_p_). To include relative maximum strength values into correlation analysis, the relative 1RM deadlift was calculated as $\frac{1\mathrm{RMdeadlift}}{\mathrm{bodyweight}\;\mathrm{of}\;\mathrm{the}\;\mathrm{participant}}$. Correlations were classified in accordance with Cohen (31): *r* < 0.2 trivial correlation, 0.2–0.5 = low correlations, 0.5–0.7 = moderate correlations and ≥0.7 = high correlations. Additionally, correlations of subgroups were evaluated for significant difference using procedure from Lenhard & Lenhard $\left(z=\;\frac{{z^{\prime }}_{1}-{z^{\prime }}_{2}}{\sqrt{\frac{1}{n_{1}-3}+\;\frac{1}{n_{2}-3}}}\right)$ (32).

## Results

**Figure 2:**
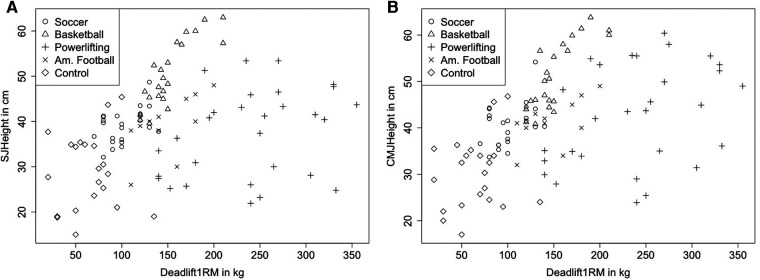
Illustrating jumping performance dependent on the deadlift 1RM considering group showing correlation coefficient of r_p_ = 0.31 (0.127–0.473, 95% CI), *p* = 0.001 in SJ (**A**) and a correlation coefficient of r_p_ = 0.462 (0.297–0.60, 95% CI) in CMJ (**B**), *p* < 0.001.

**Figure 3:**
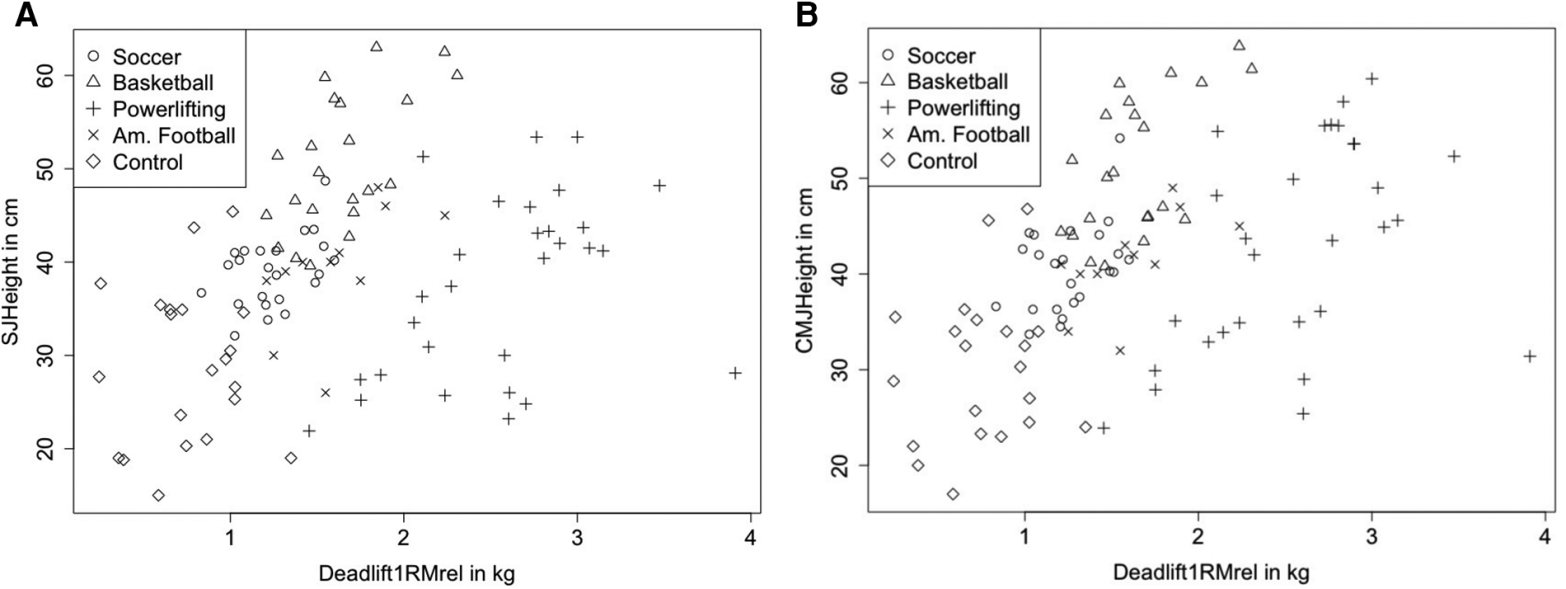
Illustrating jumping performance dependent on the deadlift rel1RM considering group showing correlation coefficient of r_p_ = 0.318 (0.135–0.479 95% CI), *p* < 0.001 in SJ (**A**) and a correlation coefficient of r_p_ = 0.466 (0.303–0.603, 95% CI) in CMJ (**B**), *p* < 0.001. Using relative 1RM deadlift, correlation coefficients showed r_p_ = 0.232 (0.029–0.417 95% CI), *p* = 0.026 for SJ and r_p_ = 0.413 (0.227–0.569 95% CI), *p* < 0.001 for CMJ.

Normal distribution of data can be assumed. Calculating the ICC for the SJ showed high reliability with ICC = 0.995 (0.993–0.997, 95% CI) and a CV of 0.21 ± 0.18%. For the CMJ, there was also high reliability with ICC = 0.995 (0.992–0.996, 95% CI) and a CV of 0.19 ± 0.16%. For the deadlift 1RM, no reliability analysis was performed as trials were performed until coordinative or conditional failure, so there were no two valid trials using the same weight. Descriptive statistics are provided in Table 1, while correlations between 1RM deadlift and relative 1RM deadlift are stated in Tables 2, 3 respectively. Figures 2, 3 illustrate the correlations between 1RM and jump performance.

**Table 1:** Descriptive statistics providing mean and standard deviation of 1RM deadlift, relative 1RM deadlift, squat jump height and countermovement jump height separated by group.

Group	Sample size	1RM deadlift (kg)	Relative 1RM deadlift	SJ (cm)	CMJ (cm)
Soccer	23	101.96 ± 20.27	1.25 ± 0.21	38.99 ± 3.74	40.62 ± 4.59
Basketball	22	152.39 ± 25.89	1.64 ± 0.29	50.58 ± 7.28	51.34 ± 7.35
Powerlifting	29	239.44 ± 66.72	2.57 ± 0.56	37.95 ± 9.76	43.62 ± 10.98
American football	11	149.09 ± 30.81	1.61 ± 0.31	37.73 ± 5.62	41.00 ± 4.80
Control	21	67.37 ± 28.55	0.79 ± 0.29	30.10 ± 7.84	31.21 ± 7.24

1RM: one repetition maximum, SJ: squat jump, CMJ: countermovement jump.

**Table 2:** Correlation coefficients r_p_ for subgroups (sports) between 1RM deadlift and jumping performance.

Group	Squat jump	Counter movement jump
Soccer	r_p_ = 0.46 (0.059–0.733 95% CI), *p* = 0.027	r_p_ = 0.437 (0.031–0.720 95% CI), *p* = 0.037
Basketball	r_p_ = 0.848 (0.665–0.935 95% CI), *p* < 0.001	r_p_ = 0.809 (0.589–0.918 95% CI), *p* < 0.001
Powerlifting	r_p_ = 0.34 (−0.055–0.613 95% CI), *p* = 0.093	r_p_ = 0.391 (−0.003–0.644 95% CI), *p* = 0.053
American football	r_p_ = 0.62 (0.039–0.891 95% CI), *p* = 0.04	r_p_ = 0.584 (−0.025–0.877 95% CI), *p* = 0.059
Control	r_p _= 0.06 (−0.382–0.479, 95% CI), *p* = 0.797	r_p_ =0.176(−0.277–0.565, 95% CI), *p* = 0.445

**Table 3:** Correlation coefficients r_p_ for subgroups (sports) between relative 1RM deadlift and jumping performance.

Group	Squat jump	Counter movement jump
Soccer	r_p_ = 0.432 (0.024–0.716, 95% CI), *p* = 0.04	r_p_ = 0.403 (−0.011–0.699, 95% CI), *p* = 0.057
Basketball	r_p_ = 0.601 (0.241–0.816 95% CI), *p* = 0.003	r_p_ = 0.572 (0.198–0.801, 95% CI), *p* = 0.005
Powerlifting	r_p_ = 0.44 (0.087–0.694, 95% CI), *p* = 0.017	r_p_ = 0.450 (0.087–0.694, 95% CI), *p* = 0.014
American football	r_p_ = 0.594 (−0.009–0.880 95% CI), *p* = 0.054	r_p_ = 0.618 (0.029– 0.888, 95% CI), *p* = 0.043
Control	r_p_ = 0.059 (−0.532–0.369, 95% CI) *p* = 0.798	r_p_ = 0.139 (−0.312–0.538, 95% CI), *p* = 0.584

### Calculation of correlations

Within the subgroups, there were significant differences in the magnitude of correlations for SJ between soccer and basketball players (*p* = 0.007) as well as powerlifters and basketball players (*p* = 0.001) and for CMJ between the same groups, favoring the basketball players in all cases (*p* = 0.02 and *p* = 0.009 respectively). In both, the SJ and CMJ, (*p* < 0.001) between basketball players showed significantly higher relationships compared to control (*p* < 0.001) while no significant difference could be observed between control and soccer players (*p* = 0.09), elite powerlifters (*p* = 0.169) and American football players (*p* = 0.059). No significant differences in magnitude of correlations could be detected between American football players and elite basketball players in SJ and CMJ (*p* = 0.092–0.14). Using correlation coefficients for 1RM or relative 1RM, there was a significant difference for SJ in basketball players and control (*p* = 0.027). The other correlations showed no significant difference between correlations measured via either 1RM or relative 1RM with jumping performance (*p* = 0.23–0.49).

## Discussion

The aim of this study was to investigate the influence of maximum strength in the 1RM deadlift on jumping performance in athletes from different sports. In accordance with the initial hypothesis, the correlations were moderated by the participants’ and athletes’ type of sport, showing the highest correlations in elite basketball players with r_p_ = 0.848. The elite powerlifters showed the lowest correlations for any athlete group with r_p_ = 0.34, while maximum strength values were the largest. Furthermore, there were significant differences in correlations between basketball players and soccer- and powerlifting athletes, as well as in the untrained participants. Since there were a variety of different sport athletes (basketball, soccer, powerlifting, American football, and athletes without prior deadlift experience), there was substantial variability in the deadlift performances.

The highest correlations between 1RM in the deadlift and both jumping types were determined in basketball players. Basketball is a high intensity sport, requiring very frequent jumping actions, but also change of direction and accelerations within a game and in training (33). McInnes et al. (34) described about 1,000 major actions within an average basketball game, including very frequent intermittent sprinting lasting up to 5 s each (52% of intermittent sprints) (33). Thus, being able to perform well in those explosive movements that occur very frequently together with jumping performance can be seen as decisive for the game outcome in basketball (10). The present results are in accordance with previous data showing high correlations between jumping and deadlift performance in elite youth basketball players (11). The American football athletes included in this study showed lower correlations than the basketball players with r_p_ = 0.62. Interestingly, American football is also stated as a sport with high demands on reaching high movement velocities, with many accelerations, decelerations and change of direction movements (35, 36). Dependent on playing position, up to 20% of the running distance is performed with sprint performance (36). To the best of the authors knowledge, no previous studies investigated the influence of 1RM deadlift and jumping performance including American football players, thus, no reference values are available. However, McGuigan et al. (37), pointed out comparable correlation coefficients between jumping performance and the 1RM squat in American football players with *r* = 0.61–0.72.

Lower correlations than in American football and basketball were obtained for soccer players with r_p_ = 0.46. While in American football, moderate running distances of about 6–8 km are reported (35, 36), in soccer literature states longer running distances in an average game with up to 12 km (38). Additionally, only 8%–12% of this distance can be assumed to be of high intensity (5). Furthermore, similar to American football, most of the high intensity activity in soccer is running performance, while jumping might be an exception for headers. Wisloff et al. (15) and Comfort et al. (17) reported higher correlation coefficients between jumping performance and the maximum strength measured via the 1RM squat than in present study. Considering a high relationship between squat strength and maximum strength in the deadlift because of similarities in the movement execution (21, 39) leading to high correlation coefficients of >0.9 (11, 25), the significant lower relationship in the present study could also be attributed to the lower performance level of the participants. While Wisloff et al. (15) included elite soccer players, participants in the present study were of moderate training level competing in the 6th soccer league in Germany. Assuming that higher competition level would lead to higher intensity within the game (40), an influence of the speed strength performance is suggested.

From a physiological point of view, maximum strength performance using complex multi-joint movements such as the deadlift as well as explosive movements like jumping performance seem to require very similar muscle activation such as maximal activation frequency, fiber recruitment and motoneuron synchronization (41). Nevertheless, based on the present results, reaching a high level of maximum strength seems not to be sufficient to increase jumping performance *per se*. Known for their very high maximum strength values, the elite powerlifters reached the highest 1RM in the deadlift with a mean of 239.44 ± 66.72 kg, but were only able to jump as high as soccer and American football players with a 1RM in the deadlift of 101.96 ± 20.27 kg and 149.09 ± 30.81 kg, respectively. Assuming that for jumping, the relative maximum strength could be hypothesized to be of higher impact, even for this parameter the power lifters outperformed the other groups with a relative 1RM of 2.57 ± 0.56 × bodyweight. In accordance with Behm & Sale (28), “Strength gains from a particular mode are not effectively transferred to another mode. Resistance training at a specific speed will exhibit optimal gains at a similar testing speed with decreasing improvements as the testing speed deviates farther from the training speed”, the results underline the high relevance of movement velocity and task specificity to transfer gained maximum strength to a particular movement. Familiarity with the movement, and therefore learning how to transfer high maximum strength values to explosive movements with a high rate of force development such as vertical jumps seem to be of high importance as the results show the highest influence of the 1RM deadlift performance on jumping performance in those athletes that incorporate jumping within their daily training routine.

Obviously, powerlifters do not need to reach high jumping heights within their sports, nevertheless this study provides important information about the relationship and transferability of maximum strength to jumping performance. These results confirm the benefits of including deadlifts to athletic training routines as an addition stimulus to train the lower body maximum strength (22) as well as increase jumping performance (26, 42). To further emphasize the results of this study, optimal jump heights are best achieved when a combination of maximum strength training such as deadlifts and squats are combined with jump training in order to integrate maximum and explosive strength as well as enhancing jump co-ordination.

No significant correlations could be detected in untrained participants. This might be attributed to the fact, that no real maximum strength could be tested in participants that have never before trained the deadlift without a significantly increased risk of injury. Consequently, it can be assumed that participants were more focused on proper movement execution instead of reaching maximum strength values (43). It can be speculated that if they would train the deadlift, maximum strength would also increase and they would get familiar with the deadlift movement execution. However, with training, those participants could not be viewed as untrained anymore.

### Limitations

Unfortunately, no athletes showing high jumping heights, but not using resistance training routines in their training routines could be included to this study, as it would be interesting to determine correlations within this group. Furthermore, American football participants had a much lower sample size (*n* = 11) compared to the other groups, so that a balanced sample size between groups could not be reached. Another limitation of this study could be seen in the different performance levels between soccer players and elite basketball players and elite powerlifters, which could influence the correlation coefficients. Therefore, 1RM testing was stopped if participants were not able to perform a deadlift with proper technique. However, this issue seems not to be addressable. The same issue could possibly contribute to comparative low correlations in soccer players, as it can be assumed that those were as well unfamiliar with testing conditions, but in this case, probably with jumping tests.

### Practical application

Results of this study confirmed high specificity and complexity of training control, which could possibly be attributed to the high specificity of the central nervous system. Even if maximum strength can be assumed to be a basic ability to reach high jumping performance, it must be considered very important to train both, the basic ability and the specific movement execution to reach a high transfer of the maximum strength to jumping performance (44). Therefore, this study underlines the importance of the combination of maximum strength training using complex multi-joint movements such as the deadlift and sport-specific movements such as jumping training to reach high performance level in the target movement.

## Data Availability

The raw data supporting the conclusions of this article will be made available by the authors, without undue reservation.
